# Health-promoting and preventive interventions for community-dwelling older people published from inception to 2019: a scoping review to guide decision making in a Swedish municipality context

**DOI:** 10.1186/s13690-020-00480-5

**Published:** 2020-10-14

**Authors:** Saranda Bajraktari, Marlene Sandlund, Magnus Zingmark

**Affiliations:** 1 Department of Community Medicine and Rehabilitation, Physiotherapy, Umeå University, Umeå, Sweden; 2 Municipality of Östersund, Health and Social Care Administration, Östersund, Sweden; 3 Department of Epidemiology and Public Health, Umeå University, Umeå, Sweden

**Keywords:** Active ageing, Healthy ageing, Nordic countries, MRC guidelines, Feasibility, Cost-effectiveness

## Abstract

**Background:**

Despite the promising evidence of health-promoting and preventive interventions for maintaining health among older people, not all interventions can be implemented due to limited resources. Due to the variation of content in the interventions and the breadth of outcomes used to evaluate effects in such interventions, comparisons are difficult and the choice of which interventions to implement is challenging. Therefore, more information, beyond effects, is needed to guide decision-makers. The aim of this review was to investigate, to what degree factors important for decision-making have been reported in the existing health-promoting and preventive interventions literature for community-dwelling older people in the Nordic countries.

**Methods:**

This review was guided by the PRISMA-ScR checklist (Preferred Reporting Items for Systematic reviews and Meta-Analysis extension for Scoping Reviews), the methodological steps for scoping reviews described in the Arksey and O′Malley’s framework, and the Medical Research Council’s (MRC) guidance on complex interventions. Eligible studies for inclusion were randomised controlled trials (RCTs) concerning health promotion or primary prevention for community-dwelling older people implemented in the Nordic countries. Additionally, all included RCTs were searched for related papers that were reporting on additional factors. Eligible studies were searched in seven databases: PubMed, SCOPUS, CINAHL, Academic Search Elite, PsycINFO, SocINDEX, and SPORTDiscus.

**Results:**

Eighty-two studies met the inclusion criteria (twenty-seven unique studies and fifty-five related studies). Twelve studies focused on fall prevention, eleven had a health-promoting approach, and four studies focused on preventing disability. All interventions, besides one, reported positive effects on at least one health outcome. Three studies reported data on cost-effectiveness, three on experiences of participants and two conducted feasibility studies. Only one intervention, reported information on all seven factors.

**Conclusions:**

All identified studies on health-promoting and preventive interventions for older people evaluated in the Nordic countries report positive effects although the magnitude of effects and number of follow-ups differed substantially. Overall, there was a general lack of studies on feasibility, cost-effectiveness, and experiences of participants, thus, limiting the basis for decision making. Considering all reported factors, promising candidates to be recommended for implementation in a Nordic municipality context are ‘Senior meetings’, ‘preventive home visits’ and ‘exercise interventions’ on its own or combined with other components.

## Background

The population across the world is growing older which calls for effective health-promoting and preventive interventions in order to help older people maintain a good quality of life. In accordance with the World Health Organisation (WHO), health promotion is defined as the process of enabling the population/individual to increase control over and improve their health, while disease prevention is defined as measures taken to prevent the occurrence of disease or limit its development [1, 2]. The implementation of health promotion and prevention is imperative given that increased levels of dependency in managing activities of daily living (ADLs) is related to a reduction in self-rated health [3] as well as higher societal costs [4]. In Sweden, municipalities have a responsibility to address health concerns and social care needs among older people ultimately aiming to optimize the person’s quality of life by promoting independence and opportunities to participate in society [5]. Therefore, municipalities need to consider health promoting and preventive interventions besides, and to complement, the provision of social care. Such interventions can promote various aspects of the health and well-being of older people by strengthening the person’s opportunities to be active and participate in society [6]. Simultaneously, a more health promoting approach to the provision of municipality services for older people could reduce the expected increase in health and social care costs.

Several studies show that health promotion and prevention in different forms have resulted in a range of positive effects such as maintenance of ability to perform ADLs [7], enhanced quality of life [8, 9], prevention of functional decline [10, 11], and reduced falls [12]. In addition, some interventions have shown to be cost-effective [13, 14]. In all, examples in the previous literature indicates that positive effects can be achieved from both multi-professional and single-professional interventions [10, 15], from both short and long-term interventions [16, 17] and both group-based and individual interventions [10, 18]. Even though the existing evidence is promising in improving health outcomes among older people, the range of interventions have varied considerably regarding their content, design and outcomes used, making them hard to compare [19]. Since resources (e.g. staff) are limited, not all promising health-promoting or preventive interventions can be implemented. Thus, more information than mere evidence on effects, based on single trials, is needed to provide sufficient guidance for decision-makers on what type of intervention to implement [20].

The question of which interventions to implement needs to be guided by a systematic decision-making process based on the best available evidence [21]. In this systematic process, a range of factors need to be considered, e.g. intervention design, effects, cost-effectiveness, feasibility of recruitment and intervention procedures as well as an understanding of how participants experience the intervention. The challenge with this task is that many health-promoting interventions often miss to report all such information relevant for decision making [22, 23]. In addition, the issue of context should be considered when assessing how evidence can be transferred from controlled trials to clinical settings [24]. In this study, the context is focused on the Nordic countries, because these countries, to a large extent, share similar welfare systems characterized by publicly funded health and social care.

A scoping review design has been proposed as an effective tool to disseminate research findings and provide an overview of evidence for decision-makers and policymakers [25], and is especially appropriate when exploring a heterogeneous or complex body of literature [26].

Given the potentially positive effects on older peoples´ health and the cost-effective use of societal resources, a comprehensive overview of the existing evidence on health promoting and preventive interventions is needed. Therefore, the aim of this review was to investigate to what degree factors important for decision-making have been reported in the existing health-promoting and preventive interventions literature for community-dwelling older people in the Nordic countries.

## Methods

This scoping review follows the PRISMA-ScR checklist (Preferred Reporting Items for Systematic reviews and Meta-Analysis extension for Scoping Reviews) [27] as well as the methodological steps for scoping reviews described in the Arksey and O′Malley’s framework [25]. The Arksey and O′Malley’s framework consists of five stages: 1) identifying the research question; 2) identifying relevant studies; 3) selecting studies; 4) charting the data; 5) collating, summarizing and reporting the results [25]. This scoping review has been conducted following an unpublished work plan.

### Identifying the research question

Health promotion and prevention often include several interacting components and can, therefore, be considered as complex interventions. The Medical Research Council’s (MRC) guidance for the process of developing, evaluating and implementing complex interventions was used to identify the research questions of this scoping review [28]. According to the MRC guidelines, this process includes several phases in which evaluations of feasibility, effectiveness and cost-effectiveness provide essential knowledge. In addition, the PICO framework (Population, Intervention, Comparison, Outcome) which is recommended to frame the research question but also to guide the whole process in a review, was used as an additional source in guiding the formulation of the research questions regarding the population, intervention/control and effects on possible outcomes [29]. Hence, the research questions were:

1. In which contexts have interventions been conducted?
2. For which populations have interventions been conducted?
3. How have the interventions been designed (e.g., which components, duration of interventions and mode of delivery)?
4. Which feasibility aspects have been described?
5. How have the participants experienced the interventions?
6. Were interventions effective, and on which outcomes?
7. Were interventions cost-effective?

### Eligibility criteria

The eligibility criteria were defined in advance but were modified with increased familiarity with the literature. Eligible studies were: 1) interventions categorised as health promotion (HP) or primary prevention (PP) following the WHO’s definition [1, 2] and addressing behavioural risk factors, injury prevention, physical health, social and mental health, 2) including populations of community-living older people 65+ as of it being the lowest retirement age in the Nordic Countries, hence exclude the risk of missing relevant studies due to the age limitation, 3) implemented in a Nordic country (Denmark, Finland, Iceland, Norway, Sweden and Faroe Islands), 4) studies applying a randomized controlled trial design (RCT) for the evaluation of effects (research question six), 5) studies related to the identified RCTs addressing the remaining research question, e.g. experiences of participants, feasibility as well as studies on cost-effectiveness. Only studies written in English were included and to decrease the risk of missing relevant articles, no year limit was applied.

The exclusion criteria were: secondary prevention programmes related to a specific disease or diagnosis e.g. interventions implemented for participants with a neurological condition such as stroke or Alzheimer’s disease, tertiary prevention programmes (e.g. rehabilitation, hospital discharge) as well as studies in populations with extensive needs for support in ADLs. Furthermore, interventions focusing on dental health promotion; interventions targeting older people with cognitive malfunction; programmes assessing effects of medication or evaluations of effects only focused on specific body structures [30], were also excluded.

### Information sources

Seven online databases were searched: PubMed, SCOPUS, CINAHL, Academic Search Elite, PsycINFO, SocINDEX, and SPORTDiscus. In designing the most suitable search strategy, a librarian at Umeå University was consulted on several occasions. The search strategy was based on a combination of words to capture key terms related to the purpose of this study: “health promotion”, “prevention”, “old people”, “community-dwelling”, “Nordic countries”, “Randomised controlled trial” and their synonyms/alternative words. A detailed outline of the search strategy, including the full syntaxes to screen the databases and numbers of search results, is available in Additional File 1. The initial search strategy was piloted and refined in the light of early findings. The search for literature was conducted from inception to January 9, 2019 (last date searched).

Identification of studies, relevant to this review, was done in two stages. At the first stage, we identified RCTs in the field of health promoting and preventive interventions for community dwelling older people conducted in the Nordic countries. To decrease the risk of missing relevant studies during the first stage of identifying studies, we did not limit our search to only primary prevention programmes. We applied this inclusion criterion when screening titles and abstracts for study selection. In the second stage, reference lists of identified and selected studies from the first stage (the RCTs) were examined for the purpose of identifying related studies, i.e. studies evaluating the same intervention but at different follow-ups, looking at different outcomes, or addressing the other research questions.

### Selecting studies

Search results were exported in EndNote reference manager, which was used to remove duplicates. In the next step, the EndNote reference manager was used to ease the process of identifying and excluding irrelevant studies through searching for key exclusion terms (hospital discharge, cognitive malfunction, dementia etc.). Titles and abstracts of the remaining studies were organised in an excel document and read independently by all authors. Studies that all authors agreed did not meet all of the eligibility criteria were removed. In cases of uncertainty, disagreement was resolved by reading the whole study and discussion among the three authors. After screening titles and abstracts and excluding studies not meeting the inclusion criteria, the remaining studies were read in full text.

### Charting the data

In line with the process of identifying research questions, the MRC framework and the PICO framework were used to guide the process of data extraction. The included studies were distributed between authors SB and MZ who independently charted the data for summarizing information related to the research questions, each question targeting one of the seven factors: context, population, intervention content, feasibility, experiences of participants, effects and cost-effectiveness. Disagreement was resolved through discussion between all authors. All authors read the extracted data and discussed the results. Main results are presented in the text under a specific heading for each of the research questions. Results are presented and described by referring to either the original study/study (At first-hand study protocol, if available. If no study protocol was identified we referred to the first published RCT), related studies (other publications related to the original study) or intervention (referring to the specific interventions evaluated in each study).

In the section below there is a description of the factors (data items) extracted to address the research questions.

### Data items

To the extent available, data on context, population, intervention content, feasibility, experiences of participants, effects and cost-effectiveness have been extracted from the included studies. The extraction of data regarding intervention context focused on identifying the setting (e.g. primary care, clinical, home, physical activities facilities) in which the specific intervention was evaluated as well as the country, and if available, the municipality in which the study was conducted. Data extracted on population concerned how the target population was defined in age, frailty/morbidities, gender, and socio-economic status. The data extracted concerning feasibility was specifically focused on identifying participation rates and retention. If a pilot or feasibility study was published, the aim and main results of the study were also extracted. Information on experiences of participants was extracted from related qualitative studies, and main results on experiences of participants were summarised.

Effects were examined by extracting effects on specific health outcomes at different time-points as reported in each study. In general, the data extracted regarding effects included effect sizes if reported, confidence intervals and *p*-values for outcomes for which a statistically significant difference was reported. No effect sizes, confidence intervals or p-values were extracted for outcomes upon which no significant difference was reported, they are mentioned in text however.

The first step in exploring cost-effectiveness was to identify if such studies had been conducted. The primary objective when looking at identified studies on cost-effectiveness was to examine if evaluated interventions were found to be cost-effective and in relation to which outcomes cost-effectiveness was established. Furthermore, if available, data concerning methodological aspects of such studies were extracted, e.g. perspective used (health provider/payer or social perspective), outcome- and cost measures and how they were affected by the specific intervention, comparator (e.g. no intervention, alternative intervention) and time horizon (over which time horizon costs and effects were measured) [31].

## Results

The search yielded a total of 690 studies. After removing duplicates, 381 titles and abstracts were screened and studies obviously not meeting the inclusion criteria were excluded. All remaining studies were read in full text (*n* = 35) and studies which did not meet the eligibility criteria were removed (*n* = 8). All 27 original studies, identified in stage 1, were in stage 2 reference checked resulting in 55 related studies being identified and included.

**Fig. 1:**
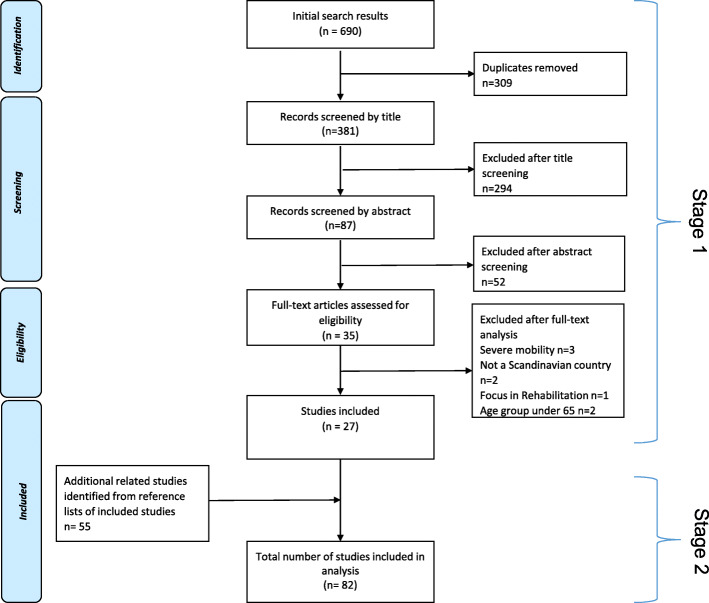
Flow diagram indicating the selection process of studies in the field of health-promoting and preventive interventions for community dwelling older people in the Nordic countries from inception to 2019

In all, a total of 82 studies were included for analysis, 27 original studies and 55 related studies. The search process is presented in a PRISMA flowchart in Fig. 1.

### Overview of original studies

The total number of participants in the included studies (extracted primarily from the original studies, if available) was 34,238. One municipality-based study included a very large sample (*n* = 24,365) [40]. Considering all studies except the one by Poulstrup and Jeune [40], sample sizes varied from 30 participants [16] to 4030 participants [53]. The duration of interventions varied from a one-session discussion group [8] to three weekly group exercise sessions over a period of one year [54]. Of the 27 original studies, 12 focused specifically on fall prevention (looking primarily at fall-related parameters and fall risk factors, e.g. falls, fear of falling, balance performance, bone mineral density) [17, 32–35, 37–41, 55]. Eight fall prevention interventions were single component and included only exercise [10, 32–36, 38, 39], while five combined an exercise component with one or more different components, e.g. preventive home visits (PHV), discussion groups, nutrition, medication review [17, 37, 40, 41, 55]. Eleven studies had a health promoting approach. Five of these studies focused on promoting general health (interventions which in addition to focusing on functional status also focused on health-related quality of life and/or social support aspects) [8, 18, 36, 43, 44], four promoted exercising [48–50, 56], and two focused on promoting mental wellbeing [16, 45]. The four remaining studies focused on preventing disability [46, 51–53]. Findings on intervention type, intervention aim, context, and population are presented below in Table 1. These findings are also described in the text, separately for each factor, in the sections below.

**Table 1:** Summary of results concerning intervention type, aim, context and population of included studies in the field of health-promoting and preventive interventions for community dwelling older people in the Nordic countries from inception to 2019

Original study ^**a**^	Intervention type	Aim	Context	Population
**Fall prevention**				
Beyer et al. 2007 [32]	Fall prevention/single component	Assess effects of a multidimensional training (resistance and balance exercise) intervention on physiological, functional and psychological conditions.	Denmark, Copenhagen Setting: gym	Women 70–90 years (*n* = 65) Inclusion and exclusion criteria: had suffered a fall that consequently required attention in an emergency room but not hospitalization, able to come to the training facility, no fractures of the lower extremities within the last six months, no neurological diseases, ability to understand Danish, a score of > 24 on MMSE.
Englund et al. 2005 [33]	Fall prevention/single component	Determine benefits of weight-bearing exercise on bone mineral density and neuromuscular function.	Sweden, Umeå Setting: Umea University, Department of Community Medicine and Rehabilitation	Women 66–87 years (*n* = 48) Inclusion and exclusion criteria: community dwelling older people, no dementia, no current smoking, no current hormone replacement therapy, not using walking aid, no cardiovascular disease, no functional disability.
Fahlstrom et al. 2018 [34]	Fall prevention/single component	Determine whether nursing assistants can prevent falls by supervising individuals with a history of falling in performing an individually designed home exercise programme.	Sweden, Örebro Setting: home-based	Older people ≥65 years (*n* = 148) Inclusion and exclusion criteria: walk independently, at least one fall during the last 12 months, able to communicate and corporate, no mental disorder, no dementia, no cancer.
Halvarsson et al. 2011 [35]	Fall prevention/single component	Evaluate effects of a progressive/specific balance training programme on fear of falling, step execution and gait, self-assessed function.	Sweden, Stockholm Setting: Krolinska University Hospital, Department of Physiotherapy	Older people 67–93 years (*n* = 59) Inclusion and exclusion criteria: self-perceived balance deficit and fear of falling, ability to walk unaided indoors, a score of ≥24 on MMSE, no severe impaired vision or hearing, no severe cancer, no severe pain, no neurological disease or damage with symptoms, no dizziness requiring medical care or heart and respiratory problems.
Helbostad et al. 2004 [36]	Fall prevention/ single component	Test effects of two exercise regimes on HRQoL and ambulatory capacity.	Norway, Trondheim Setting: home-based and group format	Older people ≥75 years (*n* = 77) Inclusion and exclusion criteria: at least one fall during the last year, use walking aid either indoor or outdoor, not exercising more than once per week, no cognitive impairment, no terminal illness.
Johansson et al. 2015 [37]	Fall prevention/multifactorial ^b^	Evaluate effectiveness/ efficacy on the experiences of participation and autonomy, risk of falls, fear of falling.	Sweden, Stockholm Setting: primary healthcare unit	Older people ≥65 years (*n* = 131) Inclusion and exclusion criteria: one or more accidental fall during the last year and/or experienced fall incidents and/or experienced fear of falling, no cognitive impairments, no psychiatric problems, no considerable difficulties in understanding and speaking Swedish.
Jorgensen et al. 2013 [38]	Fall prevention /single component	Determine motivational effects and effectiveness on mechanical lower limb muscle function, static postural balance, and functional performance.	Denmark, Aalborg Setting: Geriatric Research Clinic-Aalborg Hospital	Older people 69–81 years (*n* = 58) Inclusion and exclusion criteria: poor to average self-reported balance, no history of acute illness within the previous three weeks, no orthopedic surgery within the previous 6 months, no acute illness within the previous 3 weeks, capable of seeing visual features on the TV screen.
Karinkanta et al. 2007 [10]	Fall prevention /single component	Evaluate the specific effects of resistance training, balance-jumping training, and their combination on physical functioning and bone strength.	Finland, Tampere Setting: UKK institute Finland (The centre for health promotion research) and fitness centre	Older people 70–78 year (*n* = 149) Inclusion and exclusion criteria: clinically healthy, good self-rated physical functioning, not exercising more than twice a week, not lower than − 2.5 for the T-score for femoral neck BMD.
Kyrdalen et al. 2013 [39]	Fall prevention/single component	Compare Otago Exercise programme home training vs. group training on functional balance, muscle strength, mobility, fall efficacy, self-reported health.	Norway, 11 communities in the southeast of the country Setting: home-based and group format	Older people of mean age 82.5 (*n* = 125) Inclusion and exclusion criteria: fall prone seniors referred to a Falls Outpatient Clinic and living a maximum distance of 45 km from the hospital, a score of > 23/30 on the MMSE, able to walk without support from another person.
Palvanen et al. 2014 [17]	Fall prevention/multifactorial	Assess effects of a multifactorial intervention provided at a centre-based falls clinic on rates of falls and injurious falls.	Finland, Lappeenranta and Tampere Setting: Fall Chaos Clinic	Older people ≥70 years (*n* = 1314) Inclusion and exclusion criteria: not dementia, no terminal illness or disability which prevented physical activity and training and at least one of the following risk factors: problems in mobility and everyday function, 3 or more falls during the last 12 months, previous facture after the age of 50, an osteoporotic fracture.
Poulstrup et al. 2000 [40]	Fall prevention/multi-component ^c^	Evaluate effects of a community-based intervention on reducing numbers of fall related injuries requiring hospital treatment.	Denmark, county of Velje, five municipalities Setting: home-based and senior organizations	All older people ≥65 years from 9 municipalities (*n* = 24,365)
Sjösten et al. 2007 [41]	Fall prevention/ multifactorial	Report predictors of adherence and effects of an individually tailored intervention on health-related quality of life, incidence of falls, depressive symptoms, maximal isometric strength, postural balance.	Finland, Pori Setting: home-based and group meetings	Older people ≥65 years (*n* = 591). Sample size differs in two of the seven total related studies. Older people ≥65 years (*n* = 513) and women ≥65 years (*n* = 417). Inclusion and exclusion criteria: have fallen at least once in the previous year, able to walk 10 m’ independently.
Uusi-Rasi et al. 2012 [42]	Fall prevention/multi-component	Evaluate effects of an exercise and vitamin D intervention in reducing falls and injurious falls.	Finland, Tampere Setting: exercise halls and gyms	Women 70–80 years (*n* = 409) Inclusion and exclusion criteria: have fallen at least once in the previous years, did not use vitamin D supplements and no contradictions to exercise, were in good health and physical condition, not exercising more than 2 h per week, no regular use of vitamin D or calcium + vitamin D supplements, no recent fracture (during preceding 12 months), no contraindication or inability to participate in the exercise program, no marked decline in basic ADL, no cognitive impairments; no primary hyperthyroidism, no degenerative conditions.
**Health promotion interventions with a general health focus**				
Dahlin-Ivanoff et al. 2010 [43]	Health promotion/multi-component	Compare effects of 1) Multi-professional educational senior meetings + home visit with 2) home visit and 3) a control group on delaying deterioration, physical performance, fear of falling, physical activity, ADL, quality of life.	Sweden, Gothenburg Setting: home-based and elderly community centres	Older people ≥80 years (*n* = 459) Inclusion and exclusion criteria: at risk to develop frailty, independent in ADL, independent of home help services, cognitively intact.
Gustafsson et al. 2015 [44]	Health promotion/multi-component	Evaluate effects of a person-centred intervention on independence on ADL, self-rated health, social support, social network, loneliness, fear of falling, frailty indicators.	Sweden, Gothenburg Setting: home-based and elderly community centres	Older people ≥70 years (n = 131) Inclusion and exclusion criteria: emigrated to Sweden from Finland or Western Balkan region, independent of help from another person in ADL-staircase, no impaired cognition (scored> 80% of MMSE).
Möller et al. 2014 [18]	Health promotion/Fall prevention/multifactorial	Evaluate effects of a case management intervention on participation and leisure activities, loneliness, life satisfaction and depressive symptoms, self-reported- falls and injurious falls.	Sweden, Eslöv Setting: collaboration with municipality healthcare, social service, primary care and university hospital	Older people ≥65 years (*n* = 153). Inclusion and exclusion criteria: often in need of long-term care, dependent on ADL (two or more), admitted to hospital at least twice or have had four visits in the previous year, a score of ≥25 on MMSE, no cognitive impairment, able to communicate verbally.
Pynnonen et al. 2018 [45]	Promotion of mental wellbeing/ multi-component	Examine effects of a social intervention on depressive symptoms, melancholy, loneliness, and perceived togetherness.	Finland, Jyväskylä Setting: municipal gym, city library, health care centre	Older people 75–79 years (*n* = 257) Inclusion and exclusion criteria: feeling loneliness, melancholy or depressive mood at least sometimes, a score of > 21 on MMSE, willing to participate in the study
Rydwik et al. 2008 [46]	Health promotion/disability prevention/multifactorial	Analyse effects of a nutritional and physical training intervention on energy intake, resting metabolic rate, body composition, self-assessed function, aerobic capacity.	Sweden, Stockholm Setting: elderly research centre	Older people ≥75 years (*n* = 96) Inclusion and exclusion criteria: frail elderly defined as unintentional weight loss, low physical activity level, BMI < 30 kg/m^2^, can walk, no recent cardiac problems requiring hospital care, no hip fracture or surgery during the last six months, no current cancer treatment, no stroke within the last two year and a score of > 7 on MMSE.
Sundsli et al. 2014 [16]	Promotion of mental wellbeing/ single component	Evaluate effects of a telephone-based intervention on self-reported perceived health, mental health, sense of coherence, self-care ability, and self-care agency.	Norway, urban areas in the south of the country Setting: home-based	Older people ≥75 years (*n* = 30) Inclusion and exclusion criteria: respondents from a larger study living in urban areas in southern Norway.
Zingmark et al. 2014 [8]	Occupation focused health promotion/multi-component	Evaluate different occupation-focused interventions (individual intervention, discussion group, activity group) on leisure engagement and ADL. Evaluate cost-effectiveness.	Sweden, Umeå Setting: community meeting centre and home-based	Older people 77–82 years (*n* = 177) Inclusion and exclusion criteria: single living without home help in urban areas in northern Sweden, no cognitive or communication problems.
**Health promotion intervention with focus on physical activity**				
Kekalainen et al. 2018 [47]	Physical activity promotion/single component	Investigate effects of a supervised progressive resistance training (RT) intervention on motivational and volitional characteristics related to exercise, and if changes in these characteristics predict self- directed continuation of resistance training 1 year after the intervention.	Finland, Jyväskylä Setting: Faculty of Sport and health Sciences gym	Older people 65–75 years (*n* = 106) Inclusion and exclusion criteria: leisure-time aerobic exercise less than 3 h/wk., no previous regular RT experience, BMI < 37, no previous testosterone-altering treatment, no serious cardiovascular disease, no medication related to the neuromuscular or endocrine system, capability to walk without walking aid and non-smoker.
Niemela et al. 2011 [48]	Physical activity promotion/single component	Evaluate effects of a homebased rocking-chair intervention on physical performance.	Finland, Kauiniala Setting: home-based	Women 73–87 years (n = 51). Inclusion and exclusion criteria: females, able to follow instructions for testing and training, and informed consent to participate. Not undergoing: hip, knee, eye, stomach surgery, acute illness.
Vestergaard et al. 2008 [49]	Physical activity promotion/single component	Evaluate effects of a home-based video exercise programme on physiological performance, functional capacity and health-related quality of life.	Denmark, four municipalities Setting: home-based	Women ≥75 years (*n* = 61) Inclusion and exclusion criteria: unable to get outdoors without help from another person or walking aid, able to get out of bed or chair, able to communicate through phone, able to follow video exercises on screen, no involvement in regular physical program, not involved in regular physical activity.
Von Bonsdorff et al. 2008 [50]	Physical activity promotion/multi-component	Evaluate effects of physical activity counselling on instrumental activity of daily living and mobility limitations.	Finland, Jyväskylä Setting: primary care-based, the centre for health and social services and the department of sports and physical activity services	Older people 75–81 years (*n* = 632) Inclusion and exclusion criteria: walk 500 m without assistance, moderately physically active or sedentary (at most 4 h of walking or 2 h of exercise weekly), a score of > 21 on MMSE, no medical contraindication for physical activity.
**Disability prevention interventions**				
Lihavainen et al. 2012 [51]	Disability prevention/multifactorial	Study the effects of a comprehensive geriatric intervention on physical performance.	Finland, Kuopio Setting: gym	Older people 75–98 years (*n* = 668) Inclusion and exclusion criteria: all residents of Kuopio who were 75-years old and older, able to participate in the physical performance measures, no cognitive or physical impairment.
Luukinen et al. 2006 [52]	Disability prevention/ multifactorial	Evaluate effects of an exercise oriented intervention (home exercise, walking exercise, group activities or self-care exercise) in preventing disability and falls.	Finland, Oulu Setting: home-based or group format or in combination	Older people ≥85 years (*n* = 486) Inclusion and exclusion criteria: at least one risk factor for disability, e.g. recurrent falling during the preceding year, frequent feelings of loneliness, poor self-rated health, depression, low cognitive status, impaired vision, impaired hearing, impaired balance, slow walking speed, and impaired ability to stand up from a chair.
Vass et al. 2002 [53]	Functional decline prevention/multi-component	Evaluate effects of a community-based educational programme to home visitors and general practitioners on older people’s active life expectancy, functional ability, mortality.	Denmark, 34 communities (municipalities) Setting: primary care	Older people 75–80 years (*n* = 4060) Inclusion and exclusion criteria: citizens aged 75 year or older living in communities offering preventive home visits according to the law (2 annual visits), general practitioners should be able to participate in the preventive program, the primary care should have possibility to provide fair or good rehabilitation to citizens living in these communities.

Notes: ^a^Study protocol or the original RCT (first published RCT). ^b^Intervention components delivered to participants based on individual risk factors assessed prior to intervention. ^c^Same intervention components delivered to all participants

*Abbreviations*: *MMSE* Mini-Mental State Examination; Health related quality of life, *BMD* Bone mineral density, *ADL* Activities of daily living, *RT* Progressive resistance training, *BMI* Body mass index

### Overview of related studies

There were no related studies identified for 12 of the 27 original studies, so all 55 related studies found were linked to only 15 of the 27 original studies. Of the 15 original studies: one study reported results in nine related studies [43], two reported in seven related studies [41, 44], and one reported in six related studies [18]. The 11 remaining interventions reported results in one to five related studies. For further details, see Table 2 below.

**Table 2:** Detailed results concerning intervention content, effects on health outcomes, and feasibility aspects of included studies in the field of health-promoting and preventive interventions for community dwelling older people in the Nordic countries from inception to 2019

Original study	Related studies	Intervention content	Effects (significant between-group differences)	Feasibility aspects
**Fall prevention**				
[32]	No	**Component/s:** Moderate resistance exercise and balance exercise Modes of delivery: Groups of 5–7 participants lead by a physiotherapist **Duration:** Twice weekly (60 min) for 6 months **Control:** No intervention	**End of intervention period 6 month:** Isometric knee extension strength Newton meter (Nm) 13.5*, dynamic knee flexion (60°/180° /s, Nm) 7.2/8.1*** isometric trunk extension (Nm) 78***, isometric trunk flexion (Nm) 55***, habitual walking speed (m/s) 0.11 ***, maximal walking speed (m/s) 0.13***, Bergs Balance Scale (BBS) 1.98***. No sig. Difference in balance confidence, dynamic knee extension strength, isometric knee flexion strength, leg extension power. **One-year:** Between-group effects were maintained in most of the variables.	**Assessed for eligibility** *n* = 405 **Eligible** *n* = 261 **Randomized** *n* = 65 (I = 32; C = 33) **Dropouts:** I = 8; C = 4 No feasibility study identified
[33]	No	**Component/s:** Combined exercise program (aerobic strength, balance, coordination) **Modes of delivery:** Group sessions, led by a physiotherapist **Duration:** Twice weekly (50 min) for 1 year **Control:** No intervention	**End of intervention period 1 year:** Isometric grip strength 9,9%* higher, maximum walking speed 11,4%** higher, bone mineral density of the Wards triangle (BMD) 8,4%** higher. No sig. Difference on knee extension, one leg standing, or balance, BMD (total body, arms, lumbar spine, femoral neck, trochanter).	**Volunteers assessed for eligibility** *n* = 56 **Eligible** *n* = 48 **Randomized** *n* = 48 (I = 24; C = 24) **Dropouts:** I = 3; C = 3 No feasibility study identified
[34]	No	**Component/s:** Individually designed exercise program **Modes of delivery:** Home-based, delivered by 27 nursing assistants, 20 physiotherapists (PT) and 17 occupational therapist (OT) **Duration:** 8 home visits under 5 months **Control**: No intervention, daily calendar registration of physical exercise, walks and occurrence of fall	**End of intervention period 5 month:** BBS 2.8 points higher*, improved ADL ability*, lower bodily pain (SF-36) 13.96**, health transitions over time** (proportion with better health 10/59 in intervention vs. control 5/56). No effects for improvement of walking ability, leg strength, perceived balance, fear of falling or in health-related quality of life. **1-year:** Less hospital care due to fractures* (proportion with hospital visits due to fractures 0/59 in intervention vs. control 5/56), no sig. Difference on number of falls.	**Assessed for eligibility** *n* = 214 **Eligible** *n* = 212 **Randomized** *n* = 148 (I = 76; C = 72) **Dropouts:** I = 16; C = 16 No feasibility study identified.
[35]	[57] [58]	**Component/s:** Progressive, specific, and individually adjusted balance-training in groups **Modes of delivery:** Groups of 7–8 participants, led by two physiotherapists. **Duration:** 3 times weekly (45 min) for 12 weeks **Control:** No intervention	**End of intervention period 3 month:** Lower concern of falling (FES-I reported in median: I = 20.5 vs control C = 26)**, dual-task step execution (median: I = 1.73 vs C = 1.99)*, single task preferred walking gait in cadence (steps/min) (reported in mean I = 113 vs C = 109)*. Fast speed walking gait in velocity (m/s) (Mean: I = 1.60 vs C = 1.48)** and cadence (steps/min) (Mean: I = 1.34 vs C = 1.30)*** [35]. Improvement in overall function Cohen’s d = 0.69*, lower extremity function (basic and advanced) (Cohen’s d = 0.57* and d = 0.64*). No sig. Difference in likelihood for depression, step execution ST (initiation/step execution phase), step execution DT (initiation phase) Halvarsson et al., 2011), upper extremity function, disability (overall limitation/frequency) [57]. **9-month:** Between-group effects were maintained only in fast gait speed **, dual task step execution **, fear of falling***. **15-month:** Between-group effects were maintained only in fear of falling* [58].	**Assessed for eligibility** *n* = 146 **Eligible** *n* = 59 **Randomized** n = 59 (I = 38 and C = 21) **Dropouts**: I = 4; C = 0 No feasibility study identified
[36]	[59]	**Component/s:** Two balance/strength exercise regimens, home training (HT) versus combined training (CT) **Modes of delivery:** HT, individual self-managed home-based training and 3 group meetings with 6 participants led by physiotherapists. CT, group training with 5–8 participants led by two physiotherapists and the same home exercises. **Duration:** (HT) 2 times daily HT and 3 group meetings; (CT) 2 times daily HT and 2 times weekly classes (1 h) for 3 months.	**End of intervention period 3 month:** Mental health index (SF-36)* improved more in CT. No sig. Difference in physical health index (SF-36), walking speed (preferred/fast gait speed) [59]., functional tasks (Figure of Eight, Timed Pick-up, Sit-to-stand, Timed Up & Go, Maximum Step Length), postural sway, isometric muscle strength [36].. **9-month:** Higher number of weekly outdoor walks* and improvement in preferred walking speed only in* CT [59]. No sig. Difference in any scale of SF-36, role emotional, mental health index, physical health index, functional tasks, walking speed, postural sway, isometric muscle strength, fall rate or time to first fall [36].	**Assessed for eligibility** *n* = 127 **Eligible** *n* = 91 **Randomized** *n* = 77 (HT = 38; CT = 39) **Dropouts:** HT = 10; CT = 14 No feasibility study identified
[37]	[60] [61] [62]	**Component/s:** Group discussions on e.g., physical activity, nutrition, home safety, field visits, group and home exercise and home visits (HV) **Modes of delivery:** Group meetings of 7–8 participants, led by two therapists (occupational therapist and physiotherapist) **Duration:** 12 discussion groups (2 h) for nine months and 2 individually tailored home visits **Control:** Standard primary health care	**End of intervention period 9 month:** No sig. Difference on Perceived Participation and Autonomy Swedish version (IPA-S), perceived Occupational Gaps Questionnaire (OGQ) [37]. **12-month:** Decrease in the odds of fear of falling (FES-I) OR 0.123**. No sig. Difference in accidental falls [60], in any IPA-S domain or OGQ [37].	**Assessed for eligibility** *n* = 138 **Eligible** *n* = 137 **Randomized** *n* = 131 (I = 74; C = 57) **Dropouts:** I = 7; C = 9 No feasibility study identified
[38]	No	**Component/s:** Nintendo Wii (balance and muscle exercise) **Modes of delivery:** Sessions with 2 participants, led by a trained physiotherapist **Duration:** 2 times weekly (53 ± 5 min) for 10 weeks **Control:** Daily use of ethylene vinyl acetate (EVA) copolymer shoe insoles	**End of intervention period 10 weeks:** maximal voluntary contraction strength 8% higher (between-group difference = 269 N N)***, rate of force development (RFD) 811 N/s*, timed up and go test (TUG) -1.4 s*, fear of falling (FES-I short score) -1.2*, 30 s repeated Chair Stand Test 1.1 n**. No sig. Difference in postural balance (CoP-VM) and center of pressure velocity moment.	**Assessed for eligibility** n = 212 **Eligible** *n* = 123 **Randomized** n = 58 (I = 28; C = 30) **Dropouts:** I = 5; C = 1 No feasibility study identified
[10]	[63] [54] [64]	**Component/s:** Multicomponent exercise including resistance training (RES) or balance-jumping training (BAL) or a combination of resistance and balance- jumping training (COMB) **Modes of delivery:** Group exercise of 8–11 participants in RES and COMB, 17–21 participants in BAL, led by an exercise leader **Duration:** 3 times weekly (50 min) for 1 year **Control:** No intervention	**End of intervention period one year:** Self-rated physical functioning (Rand 36-items health survey) improved 10% more in COMB vs. control, dynamic balance (figure-of-8 running time, s) improved more in BAL and COMB vs. RES (6 and 8% respectively), leg extensor force (Leg press, N/k) improved more in RES and COMB vs. control (14 and 13% respectively), tibial shaft bone strength index (BSI, mm^3^) decreased 2% less in COMB vs. control, femoral neck in in section modulus in RES vs. COMB showed 4% higher treatment (Z, mm^3^). No sig. Difference in bone health parameters: bone mineral content (BMC) at proximal femur, distal tibia, distal radius, radial shaft [10]. No sig. Difference in health-related quality of life (HRQoL), fear of falling (FoF) [54]. **1-year:** Improvement in dynamic balance remained in COMB vs. control (4%), tibial shaft bone strength preserved 2% benefit in COMB vs. control. No effects remained in self-rated physical functioning, leg extensor force, section modulus (Z) at the femoral neck [63], HRQoL, FoF [54]. **6-year:** Rate of injured fallers was 62% lower in COMB HR 0.38 vs all, COMB group had 51% less injurious falls RR 0.49 and 74% less fractures RR 0.26 vs control, RES, BAL [64].	**Assessed for eligibility** *n* = 241 **Eligible** *n* = 166 **Randomized** *n* = 149 (RES *n* = 37, BAL n = 37, COMB *n* = 38, C = 37) **Dropouts:** RES = 0; BAL = 2; COMB = 2; C = 1 No feasibility study identified
[39]	No	**Component/s:** Otago exercise program (Balance and muscle strengthening program), group-based (GT) vs. home-based (HT) **Modes of delivery:** Groups of 4–5 participants, led by a physiotherapist or independent home-training **Duration:** 2 times weekly group training (45 min) or 3 times weekly home training (30 min) supported with 4 visits from a physiotherapist over a period of 12 weeks	**End of intervention period 3 months:** BBS 3.2 points higher*, improvement in Sit-to-Stand test (STS) 2.2 * and Physical Health Index (SF-36-PH) 45 (0 − + 100) ** in favor of GT. No sig. Difference in mobility (Timed Up-and-Go), mental health (SF-36-MH), fall efficacy (FES-I). **6-month:** Improvement STS 2.2 (number of trials)** and TUG − 2.4 s* in favor of GT. No sig. Difference in BBS, falls efficacy, physical/mental health index.	**Assessed for eligibility** *n* = 205 **Eligible** *n* = 171 **Randomized** n = 125 (GT = 62; HT = 63) **Dropouts:** GT = 22; HT = 20 No feasibility study identified
[17]	No	**Component/s:** Multifactorial intervention including: strength and balance training, medication review and referrals, proper nutrition, home hazard assessment and modifications of home environment **Modes of delivery:** Baseline assessment and intervention implementation was carried out as appropriate by a nurse, physician, and physiotherapist **Duration:** Each participant received on average 5 interventions or recommendations over 1 year **Control:** Received a general injury prevention brochure of the Finnish Prevention of Home Accidents Campaign	**End of intervention period 1 year:** Falls rate was lower in the intervention group (95 falls per 100 person-year) vs. control (131 falls per 100 person-year) (IRR = 0.72, 95% CI 0.61–0,86)***, ratio of fallers was 22% lower in I compared to C at any time point during the intervention (HR = 0.78, 95% CI 0.67–0.91)***. Fall-induced injuries for I was *n* = 351 compared to the C *n* = 468 (IRR = 0.74, 95% CI 0.61–0.89)**. No sig. Difference in the number of fractures.	**Assessed for eligibility** *n* = 1601 **Eligible** *n* = 1570. **Randomized** *n* = 1314 (I = 661; C = 653). **Dropouts:** I = 72; C = 97 No feasibility study identified
[40]	No	**Component/s:** Community-based intervention consisting of Information, treating somatic and psychiatric illnesses, and dealing with improper drug consumption, diet insufficiencies and physical and mental inactivity and home visits with follow-up, removing physical hazards in the home **Modes of delivery:** Leaflets and talks on clubs for senior citizens and home visits to people, 70–74-year-old, by utilizing existing municipal personnel e.g. nurses or practitioners, home helpers **Duration:** 18 months. Training was delivered once in the beginning of the intervention and once halfway through **Control:** Four other municipalities offering the standard healthcare	**End of intervention period 18 month:** Reduction of lower extremity fractures in the IG by 33% (OR = 0.63, 95% Cl 3–63)*. In women, the reduction of lower extremity fractures was 46% (95% Cl 8–84)* whereas in men, there was no sig. Effect. No sig. Difference reduction in the number of all fractures.	**Study population** *n* = 26,221 (I = 13,921 and C = 12,300) **Randomized** n = 26,221 (I = 12,905 five municipalities; C = 11,460 four municipalities) **Dropouts:** not reported. No feasibility study identified
[41]	[65] [66] [67] [68] [69] [70] [71]	**Component/s:** individual geriatric assessment, Individual guidance on fall prevention, physical exercise in small groups, lectures, psychosocial group activities, home exercise, home hazards assessments **Modes of delivery:** Individually based, home-based and group sessions. Led by health professionals, student nurse, public health nurse. **Duration:** 1 occasion of geriatric assessment, 1 occasion of oral and written information, 2 times monthly group exercise of 4–10 participants (about 50 min), 1 time monthly lectures on preventive aspects of falling, 1 time monthly psychosocial activities organized in two groups, those with lower social contacts and scores over 10 on Geriatric Depressive scale joint a smaller support group, the rest joined a bigger group, 1 time weekly home exercise for one year, 1 home hazard assessment in the beginning of the intervention and 1 six month after the intervention period. **Control:** 1 counselling session on fall prevention at baseline	**End of intervention period 1 year:** Sig. differences only in women (I vs C): Velocity moment in standing balance decreased with a median change of − 0.54 mm^2^/s* [65], improvements in usual activity (cumulative odds ratio (COR) 1.4, CI 95% 1.0–1.8)** and in discomfort/symptoms (COR 1.4, 95% 1.1–1.8)* [66], extension strength of the left/right knee increased 5% /3%** more in I vs C [68].Sig. differences only in men (I vs C): Depressive symptoms decreased in I vs control only in men with a mean difference of − 2.5** [71]. Improvement on some dimensions of health-related quality of life: depression (COR 10.1, 95% 1.5–67.0)* and distress (COR 5.6, 95% 1.6–19.3)* [66]. Falls incidence decreased in those with higher number of depressive symptoms IRR = 0.50**, 95% CI = 0.92–1.57 and vice versa, in those with at least three previous falls IRR = 0.59**, 95% CI = 0.38–0.91, in subjects with high perceived risk of falling IRR = 0.77*, 95% CI = 0.55–1.06 [68]. No sig. Difference in hand grip strength, knee flexion (right/left) [68], incidence of falls overall [71] or in the incidence of falls requiring medical treatment [69], depressive symptoms [70], dynamic balance [65]. **2-year and 3-year:** No sig. Difference between I vs control in the incidence of falls requiring medical treatment [69]..	**Assessed for eligibility** *n* = 612 **Eligible** *n* = 591 **Randomized** n = 591 (I = 293; control = 298) **Dropouts:** I = 32; control = 29 No feasibility study identified
[42]	[72] [73] [74] [55]	**Component/s:** Vitamin D and exercise combinations consisting of No exercise + Placebo (D-Ex-) or No exercise + vitamin D (D + Ex-) or Exercise + Placebo (D-Ex+) or Exercise + vitamin D (D + Ex+) (Uusi-Rasi et al., 2015) **Modes of delivery:** Groups of 5–10 participants and home exercises, led by 1 or 2 exercise leaders **Duration:** 2 times weekly exercise sessions (60 min) for the first year, 1 weekly exercise sessions (60 min) during the second year, including maximum 20 participants, and home exercise (5–10 min) on days without groups exercises during the first year and at least 3 time per week during the second year	**End of intervention period 2 year:** Leg strength (mean change: 14.1, 95% CI = 8.0–20.2 in exercisers; 1.6, 95% CI = -4.5 to 7.7 in no exerciser regardless vitamin D or placebo group) ***. Chair stand time also differed between groups (7.4, 95% CI = 3.8–10.8% in exercisers; 2.4, 95% = CI − 1.6-6.2 in no exerciser regardless vitamin D or placebo group) **. Neither exercise nor vitamin D reduced falls. Fall rates per 100 person-years were 118.2, 132.1, 120.7, and 113.1 in the D-EX-, D + EX-, D-EX+, D + EX+. Injurious fall rates were 13.2,12.9,6.5, and 5.0, respectively. Hazard ratios for injured fallers were lower among D + EX+ (HR = 0.38, 95% CI = 0.17–0.83) and D-EX+ (HR = 0.47, 95% CI = 0.23–0.99). Irrespective of vitamin D exercise improved muscle strength (mean increase in lower limb extension strength almost 15%)***, D-EX+ improved more than 6% in Chair stand test*. Vitamin D maintained femoral neck bone mineral density. No sig. Differences in TUG, grip strength, total falls incidence rate ratio [74]. **4-year:** All treatment groups had less medically attended injurious fallers (HR = 0.62, 95% CI 0.39–1.00 for D + EX-), (HR = 0.46, 95% CI 0.28–0.76 for D-EX+) and HR = 0.55, 95% CI 0.34–0.88 for D + EX+) compared with D-EX-. Leg extensor muscle strength (N/kg) remained about 10% higher in D-EX+ and about 12% higher in D + EX+ vs D-EX- (Uusi-Rasi et al., 2017). Isometric leg extension strength improved in exercisers with a mean difference of 12.5%***, chair stand time reduced in exercisers with mean difference of 5%**, fast walking speed improved with 4.3%** in exercisers vs controls, greater probability in exercisers to complete backward walking test vs control (6.1 m)*** (74.3% of exercisers vs 48.8% control) [74].	**Assessed for eligibility** *n* = 1213 **Eligible** *n* = 433 **Randomized** *n* = 409 D-Ex- (*n* = 102), D + Ex- (n = 102), D-Ex+ (*n* = 103), D + Ex+ (n = 102) **Dropouts:** D-Ex- =7; D + Ex- =14; D-Ex+ =12; D + Ex+ = 6 No feasibility study identified
**Health promotion interventions with a general health focus**				
[43]	[75] [76] [77] [78] [79] [15] [80] [81] [7]	**Component/s:** Senior group meetings (SM) and 1 follow-up home visit or a single preventive home visit (PHV) **Modes of delivery:** Senior group meetings with a maximum of 6 participants or a single home visit led either by an occupational therapist, physiotherapist, registered nurse or social worker **Duration:** Weekly SM sessions (2 h.) for 4 weeks+ 1 PHV or a single PHV. **Control:** Access to the ordinary range of services for older persons provided by the urban district	**3-month:** SM vs. control: postponed dependence in activities of daily living (ADL) OR 1.95 **. PVH vs. control: delayed deterioration in self-rated health OR 2.21*. All vs control: no sig. Difference in frailty [7], functional balance, walking speed, physical activity, falls efficacy [77]. **1-year:** SM vs. control: positive effect on social support (regarding someone to turn to when in need of advice and backing) OR 1.72** [79], postponed independence in ADL OR 1.92** [81], delayed deterioration in self-rated health OR 0.55* [78], larger physical activity performance OR 1.82* [77], delayed deterioration in morbidity OR 0.61* [78], maintained satisfaction with physical health OR 0.57* [78]. PVH vs. control: larger physical activity performance OR 1.99* [77], delayed deterioration in morbidity OR 0.44** [78], maintained satisfaction with physical health OR 0.49* [78]. All vs. control: lower odds of dissatisfaction with psychologic health OR 0.34**/0.45* [78]. No sig. Difference on loneliness, social network, or other aspects of social support [79], walking speed, falls efficacy [77], frailty [15]. **2-year:** SM vs. control: larger physical activity performance OR 1.73* [77], higher odds of having a total score of 48 or higher on BBS OR 1.96** [77], lower concern of falling Short Falls Efficacy Scale International (FES-score)a (9.0 vs 13.0)*, delayed deterioration in morbidity OR 0.52*, maintained satisfaction with physical health OR 0.28** [78]. PVH vs. control: larger physical activity performance OR 2.10** [77], higher odds of having a total score of 48 or higher on BBS OR 1.80** [77], lower concern of falling (FES-score), (11.1 vs 13.0)*, delayed deterioration in morbidity OR 0.60* [78], lower odds of dissatisfaction with physical health OR 0.43** [78]. All vs. control: lower odds of dissatisfaction with psychologic health OR 0.40**/0.30*** [78]. No sig. Difference in ADL, self-rated health, frailty, mean walking speed.	**Assessed for eligibility** *n* = 546 **Eligible** *n* = 491 **Randomized** *n* = 384 (PVH = 174; SM = 171; **Control** *n* = 39) **Dropouts:** PVH = 35; SM = 38; C = 19 No feasibility study identified
[44]	[82] [83] [84] [79] [85] [86] [87]	**Component/s:** Senior group meetings (SM) discussing different aspects of self-management of health and a follow-up home visit **Modes of delivery:** Groups of 4–6 participants and one follow-up home visit, led by a multidisciplinary team **Duration:** Weekly SM sessions for 4 weeks **Control:** No intervention	**6-month:** Increase in the total score of sense of coherence (SOC-13) (OR = 2.23, 95% Cl 1.05–4.77)* [86]. No sig. Difference in maintaining independence in ADL, maintaining/improving self-rated health [84]. **1-year:** Positive effects on social support (having someone to turn to when in need of advice and backing) OR 1.72** [79]. No sig. Difference in SOC [86], maintaining independence in ADL, maintaining/improving self-rated health [84], on loneliness, social network, or other aspects of social support (e.g. having someone to trust and confide, to turn to for practical help) [79].	**Assessed for eligibility** *n* = 873 **Eligible** *n* = 779 **Randomized** *n* = 131 (I = 56; C = 75 **Dropouts**: I = 9; C = 13 [84]. Pilot study assessing the feasibility of an adapted protocol of Senior meetings from “Elderly in the risk zone” [43]
[18]	[88] [89] [90] [91] [92] [93]	**Component/s:** Case management comprised of four dimensions: Case management tasks, General information, Specific information, Safety and continuity. **Modes of delivery:** Individually home-based, including a care plan monitored by case managers (2 nurses and 2 physiotherapists) and a physical training program performed by the participant **Duration:** At least 1-time monthly home visit for 1 year **Control:** One year waiting list to get the intervention	**3-month:** Intervention group performed leisure activities in general to a greater extent than the control group (median: number of activities n = 13 vs n = 11). No sig. Difference on social participation [89]. **6-month:** Complete case analysis: risk for depressive symptoms RR = 0.49* and life satisfaction ES = 0.41* in the intervention group vs. control. **6 months to 12 months:** Intention to treat analysis: no sig. Difference in loneliness, life satisfaction, depressive symptoms [88].. Fewer emergency department visits not leading to hospitalization in the intervention group vs. control (mean: 0.08 vs 0.37)*. Fewer visits to physicians in outpatient care (mean: 4.09 vs. mean 5.29)* [93]. **End of intervention period 1 year:** Intention to treat analysis: no sig. Difference in depressive symptoms [88]. Complete case analysis: sig. Difference in depressive symptoms ES = 0.47* [88]. No sig. Difference in preventing falls or injurious falls [18], life satisfaction, loneliness [88].	**Assessed for eligibility** *n* = 1079 **Eligible** *n* = 848 **Randomized** *n* = 153 (I = 80; C = 73) **Dropouts**: I = 21; C = 15 Pilot study aiming to test sampling and sample characteristics
[45]	No	**Component/s:** Social intervention of choice (exercise program-EP or social activity program-SP or personal counseling-PC) **Modes of delivery:** Group meetings for the EP and SP program. EP was led by qualified instructors at the municipality gym, SP was led by healthcare students in the city library, PC was led by a rehabilitation counselor in a healthcare center **Duration**: Weekly sessions for the EP and SP program (altogether 19–21 times). PC every third week, 4–5 meetings per participant (during the 6-month intervention) **Control:** One counseling session	**End of intervention period 6 month:** Social integration increased in I (EP + SP + PC analyzed as one single intervention) but not in control (Generalized estimating equations-GEE group x time 0.041) *. No sig. Difference in Attachment and guidance, feelings of loneliness and melancholy, depressive symptoms. **12-month:** No sig. Difference in loneliness and melancholy.	**Assessed for eligibility** *n* = 985 **Eligible** *n* = 475 **Randomized** *n* = 257 (I = 129; C = 128) **Dropouts:** I = 24; C = 10 No feasibility study identified
[46]	[94] [95] [96]	**Component/s:** Individual nutritional advice and group sessions on nutrition (N) or physical training (T) or combined nutrition and physical intervention (N + T) **Modes of delivery:** Individually based and group sessions. (T) led by a physiotherapist and trained instructor, (N) led by dietitians **Duration:** (T) 2 times weekly group meetings (1 h.), (N) 1 individual counseling and 5 group sessions for 3 months **Control:** General physical training advice and general dietary advice	**End of intervention period 3 month:** Resting metabolic rate (RMR) varied in T with a mean difference of 4.8 Mj/d* [96]. Leg press improved in T + N and T vs N (mean difference: 11.4 kg respective 14.3 kg)*. Improvement in dips in T + N and T vs C (mean diff: 2.9 kg respective 3 kg)**, improvement for step test in T vs T + N 4.3* [46]. Lower extremity muscle strength increased in T and T + N vs N (mean diff: 87 kg respective 81 kg). Activity level increased in T and T + N vs C (median level: 3 vs 3)*. Walking duration increased* for combined (T and T + N) vs N and C [94]. No sig. Differences in balance, mobility, nutritional measures (e.g. body weight, energy intake) [46, 96], aerobic capacity (maximal work-load or work time) [95]. **9-month:** Only effects in physical activity level preserved in T vs C and N [94]. No effects were preserved on: RMR, leg press, dips, step test, muscle strength [46], Aerobic capacity (maximal work-load or work time) [95], ADL [94].	**Assessed for eligibility** *n* = 2012 **Eligible** *n* = 672 **Randomized** *n* = 96 (N n = 25, T *n* = 23, N + T n = 25, C = 23) **Dropouts:** T = 3; *N* = 3; N + T = 7, C = 4 No feasibility study identified
[16]	No	**Component/s:** Telephone calls focused on self-care habits, eating habits and nutrition, physical activity, health promotion, identity and self-esteem and one meeting **Modes of delivery:** Telephone calls following a single meeting with all health professionals involved in the intervention (two occupational therapists and one physiotherapist) **Duration:** Five self-care telephone calls (30 min) over a period of 19 weeks **Control:** No intervention	**End of intervention period 19 week:** Mental health GHQ-30 (Goldberg’s General Health Questionnaire) improved in I with 4 scores vs C who experienced a decrease with 4 score*. No sig. Difference in Self-Care Ability scale for the elderly, Appraisal of self-Care Agency, sense of Coherence.	**Assessed for eligibility** *n* = 1044 **Eligible** *n* = 284 Randomly chosen sample *n* = 226 Answered baseline questionnaire (I = 15 city A; C = 64 city B) **Randomized** (those who answered baseline questions): (I = 15 city A; C = 15 city B). **Dropouts:** I = 0; C = 0 No feasibility study identified
[8]	[13]	**Component/s:** Three different occupation-focused interventions: individual intervention (IG) or activity group (AG) or discussion group (DG) **Modes of delivery:** Home-based and telephone calls (IG), or group sessions in the format of a discussion group (DG) or an activity group (AG). Led by experienced occupational therapists **Duration:** IG three to eight contacts either as home visits or telephone calls. AG 8 weekly sessions of 5–8 participants (1,5 h.), DG one 2 h-meting including 7–9 participants **Control:** No intervention	**3-month:** DG had a small effect on reducing decline in leisure engagement compared to the control group. (Cohen’s d 0.27). The IG and DG had a small effect in maintaining ADL ability (Cohen’s d 0.29 and 0.31 respectively) [8]. AG and DG had a positive effect on self-rated health *(Zingmark et al., 2015. **12-month:** IG had a small effect on reducing decline in leisure engagement (Cohen’s d 0.41). IG, AG and DG had a small effect in maintaining ADL ability (Cohen’s d 0.30, 0.38 and 0.30 respectively) [8]. No intervention had an effect on self-rated health at 12 months.	**Assessed for eligibility** *n* = 680 **Eligible** *n* = 549 **Randomized** *n* = 177 (IG = 41, AG = 49, DG = 41, C = 46) **Dropouts:** IG = 1; AG = 1; DG = 6; CG = 4 No feasibility study identified
**Health promotion intervention with focus in physical activity**				
[47]	No	**Component/s:** Resistance training (RT). **Modes of delivery:** Group training with different intensity, led by trained personnel **Duration:** Twice weekly resistance exercise for all groups (1 h) for the 3 first months. Allocated frequencies during 4th to 9th month. Group 1 (RT1) exercised once weekly, group 2 (RT2) twice weekly group 3 (RT3) three times weekly. **Control:** No exercise	**3-month:** Improvements in exercise self-efficacy, coping planning (group × time)*, intrinsic motivation to training (group × time)** No sig. Difference in other volitional or motivational parameters (action planning, external/introjected/identified/intrinsic motivation to training or physical activity). **3 to 9 months (end of intervention period):** No sig. Difference in any motivational or volitional parameters. **Baseline to 9 months:** Action planning improved in all groups vs. control***. Coping planning and intrinsic motivation related to physical activity improved in RT2 and RT3 vs. control*. Intrinsic motivation related to training improved in RT2 and RT3 vs RT1 and control***. **12-months:** 54% of participants did not continue self- directed regular resistance training, 22% continued regular resistance training once- a- week, and 24% twice- a- week.	**Assessed for eligibility** n = 454 **Eligible** n = 148 **Randomized** n = 106 (RT1 n = 26; RT2 *n* = 27; RT3 *n* = 28; C = 25) **Dropouts:** RT1 = 1; RT2 = 2; RT3 = 0; C = 2 No feasibility study identified
[48]	No	**Component/s:** Rocking-chair training program (RCG) and 10 different movements **Modes of delivery:** Home-based exercise **Duration:** Twice daily sessions (15 min) for 6 weeks **Control:** No intervention	**End of intervention period 6 weeks:** BBS score (Mean: 51.5 vs 49.4)***, maximal knee extension strength (N) (mean: RCG 266.1 vs CG 225.9)**, maximal walking speed (m/s) (mean: 1.4 vs 1.4)* in favor of RCG. No sig. Difference in standing on one leg, hand grip, or chair rise parameters.	**Assessed for eligibility** *n* = 112 **Eligible** *n* = 97 **Randomized** n = 51 (I = 26; C = 25) **Dropouts:** I = 1; C = 1 No feasibility study identified
[49]	No	**Component/s:** Aerobic and strengthening exercise **Modes of delivery:** Home-based video-exercise (consisting of a videotape showing the exercises, booklet describing the exercises and an elastic resistance band) and bi-weekly telephone calls, an exercise instructor assisted the first training session **Duration:** three times weekly (26 min) for 5 months **Control:** no intervention, received same telephone calls as the intervention group	**End of intervention period 5 months:** Improvement in EQ-5D in I vs C (mean: 0.77 vs 0.64) **. No sig. Difference in physiological measures e.g. handgrip strength, biceps strength or functional ability measures e.g. maximal walking speed, physical performance, self-rated health (S-R health). Significant within group improvement ranging from 8 to 35% in physical performance test, mobility, handgrip, biceps strength, chair rise, 10 m maximal walking speed.	**Assessed for eligibility** *n* = 650 women **Eligible** *n* = 454 **Randomized** *n* = 61 (I n = 30; control *n* = 31) **Dropouts:** I = 5; C = 3 No feasibility study identified
[50]	[97] [98]	**Component/s:** Physical activity counselling Modes of delivery: Individually based telephone calls by a physiotherapist **Duration:** One single individual motivational face-to-face physical activity counseling sessions and phone calls every 4 months from a physiotherapist for 2 years **Control:** No intervention	**End of intervention period 2 years:** Perceived difficulty in advanced mobility (walk 2 km) was lower in the I group vs. control (OR = 0.84, 95% CI 0.70–0.99)* [97]. Higher proportion of participants in I vs control increased activity level from sedentary till at least moderate (83% vs 72%, OR 2.0, 95% CI: 1.3–3.0). Lower proportion of participants in I vs control reduced their physical activity level from at least moderate to sedentary (17% vs 28%, OR 0.51, 95% 0.3–0.8) [97]. No sig. Difference on basic mobility (walk 0,5 km) [97], IADL disability (preparing meals, washing clothes, shopping and more) [50].. Subgroup analysis (according to IADL status at baseline): intervention resulted in a reduced incidence disability in those without disability at baseline (RR = 0.68, 95% CI 0.47–0.97)*, no sig. Difference for IADL disability. **3.5-year:** Treatment effect on perceived difficulty in advanced mobility preserved (OR 0.82, 95% 0.68–0.99)*.	**Assessed for eligibility** *n* = 1310 **Eligible** *n* = 1040 **Randomized** *n* = 632 (I = 318; C = 314) **Dropouts:** I = 23; C = 31 No feasibility study identified
**Disability prevention interventions**				
[51]	[99] [100]	**Component/s:** Multidisciplinary check-ups, physical activity counselling and supervised strength and balance training **Modes of delivery:** Individual multidisciplinary check-ups led by a physician, nurse and physiotherapist and one-time group-based muscle strength and balance training and once weekly resistance training **Duration:** one annually physical activity counselling (75 min, including 15-min warming-up and balance exercise), one opportunity to participate in supervised strength and balance training and once weekly resistance training for 2 years **Control:** no intervention, annual healthcare and physical performance evaluation	**End of intervention period 2 year**: BBS 1.13 points higher***, maximal walking speed 0.05 m/s better***, TUG 0.97 s quicker in completing the test*** [51]. Improved chair rise capacity in physically active women with − 1.67 s*. No improvement in inactive women or in men, regardless of their physical activity level [99]. The intervention prevented the loss of ability to walk 400 m among pre-frail and frail persons OR 0.74** (95% CI 0.59–0.93). The treatment effect was not significant among non-frail participants [100]. **3-year:** Between-group effects were maintained in balance, maximal walking speed, and TUG [51].	**Assessed for eligibility** *n* = 1000 (random sample) **Eligible** *n* = 1000 **Randomized** *n* = 928 (Active = 461; inactive = 467) [99] **Dropouts:** I = 197; C = 221 No feasibility study identified
[52]	[101]	**Component/s:** exercise program consisting of home exercise or walking exercise or group exercise or self-care exercise or in combination **Modes of delivery:** delivered based on individual risk factors planned and assessed prior intervention by a regional geriatric team (physiotherapist, occupational therapist, physician) **Duration:** home exercise recommended to be conducted three times per day (5–15 repetitions) for 1 year and 4 months or group exercise in small groups or self-care exercise. **Control:** Were asked to visit their family physicians without a written intervention form	**End of intervention period 16 months:** Mobility performance improved in favor of home exercise group (median 0.5, interquartile range 0–2.0)* [52]. Impaired balance less common in intervention vs control (*n* = 64; 45% vs *n* = 89; 59%)* [101]. No sig. Difference in admission into long-term institutional care, severe mobility restriction, ADL [52]. Falls and time to first four falls [101].	**Assessed for eligibility** *n* = 555 **Eligible** *n* = 486. **Randomized** n = 486 (I = 243, C = 243) **Dropouts**: I = 37; C = 65 No feasibility study identified
[53]	[102] [103] [104] [105] [106]	**Component/s:** Regular education and a short assessment program for healthcare personal (home visitors) providing standard preventive home visits in 17 Danish municipalities (two annual home visits to all citizens aged 75 years or older). Education consisted of emphasizing the importance of psychological, social as well as health factors, focusing on early signs of disability, empowering strategies and social relations with respect to the individual’s autonomy, stressing the importance of physical activity and focusing on relevant geriatric problems. **Modes of delivery:** Group education program **Duration:** Regular education (17 municipal meetings) for home visitors during 3 years and one education programme (2 h) for GPs in the first year **Control:** No intervention (education program) for home visitors in another 17 control municipalities.	**End of intervention period 3 year:** Reduction in functional decline (among 80-year-olds) in intervention municipalities I vs control municipalities C, OR 1.83**, 95% CI 1.21–2.77. Intervention in coordination with GP was related to better functional ability only for women OR 1.26**, 95% 1.08–1.47. Accepting and receiving preventive home visit was also related with improved functional ability only in women OR 1.36**, 95% CI = 1.16–1.60 [105]. No sig. Difference on Nursing home admission or mortality [103]. **4,5-year:** Lower risk for progressive decline in intervention municipalities vs control RR 0.66**, 95% CI 0.50–0.86. In participants who declined home visits was related with increased risk for catastrophic functional decline RR 1.49***, 95% CI 1.27–1.74 [106]. Fewer persons (80-year-olds) in the intervention group had moved to a nursing home HR 0.59*, 95% CI 0.37–0.94 [103]. Effects on functional ability in women were preserved OR 1.22*, 95% CI 1.03–1.44. No sig. Difference in functional ability for men [104]. No sig. Difference in functional decline or mortality in both man and women [103].	**Eligible** *n* = 5788 (invited participants) **Randomized** *n* = 4034 (I municipalities = 2092; control municipalities = 1942) **Dropouts:** I = 31; C = 27 [105] No feasibility study identified

Notes: *p ≤ 0.05, **p ≤ 0.01, ***p ≤ 0.001. ^a^Maximum score for FES-1 = 28, higher score implies higher concern for falling, lower score implies lower concern for falling, ^b^Maximum score for BBS = 56, higher score implies higher degree of functional balance and vice versa, ^c^Maximum score for 13-item Orientation to Life Questionnaire (SOC-13 = 91), higher score indicates high SOC and vice versa

*Abbreviations*: *OR* Odds Ratio, *IRR* Incidence Rate Ratio, *HR* Hazard Ratio, *RR* Risk Ratio, *BBS* Bergs balance score, *FES-I* Falls Efficacy Scale, *SF-36* Short Form Health Survey, *IPA-S* Perceived Participation and Autonomy Swedish version, *OGQ* Occupational Gaps Questionnaire, *RFD* Rate of Force Development, *HRQoL* Health-Related Quality of Life, *FoF* Fear of Falling, *SF-36-PH* Physical Health Index, *SF-36-MH* Mental Health Index, *STS* Sit-to-Stand test, *TUG* Timed Up-and-Go; *SOC-13* Sense of Coherence score, *RMR* Resting Metabolic Rate, *GHQ-30* Goldberg’s General Health Questionnaire, *IADL* Instrumental Activities of Daily Living

Among the 55 related studies, 38 included evaluations of effects, eight were qualitative studies analysing experiences of participants, four were health economic evaluations, three were study protocols, and two were pilot studies. Findings on intervention content, effects and feasibility aspects are also described separately in the sections below, while detailed information on these factors is presented in Table 2.

### Intervention context

Geographically, the studies were conducted in Finland (*n* = 10), Sweden (*n* = 9), Denmark (*n* = 5) and Norway (*n* = 3). No studies were identified from Iceland or Faroe Islands. Interventions were implemented either at home (*n* = 4) or in other settings (*n* = 11), e.g. gyms and exercise halls [32, 42, 47, 51], clinics/hospitals [17, 37, 38, 50, 53] or research centres [10, 46]. The remaining interventions were implemented in a combination of settings (*n* = 12). For further details, see Table 1, “Context” column.

### Population

The population targeted in the included studies varied regarding age and health-related conditions. In six studies, the target population was defined in relation to age and location of residence [16, 33], four of these studies were municipality-based and targeted a broad population of older people from several municipalities [40, 45, 53, 107]. The remaining studies defined the target population in relation to age and location of residence/municipality in combination with criteria related to general health or frailty (*n* = 21), e.g. fall-related reasons such as having a fall or experienced fear of falling. The mean age across studies ranged from 65 years to 93. Twenty of the studies included participants above a certain age, i.e. 65 years or older (*n* = 6), 70 years or older (*n* = 7), 75 years or older (n = 7), 80 years or older (*n* = 1), and 85 years or older (n = 1). Four studies applied a broad age span, e.g. 66–87 years [33], 67–93 years [35], 69–81 [108], 73–87 [48], whereas two applied a narrow age span 77–82 [8], 75–79 [45]. One study reported only the mean age of the participants [39]. Five studies had samples consisting only of female participants [32, 33, 42, 48, 49]. For further details, see Table 1, “Population” column.

### Intervention content

Given the broad range of intervention types, interventions varied by content, modes of delivery, duration and professionals involved. In most of the studies, the intervention content included a physical activity component (*n* = 19). In twelve of these studies, exercise was the only component and included different exercise forms such as resistance/strength [47], balance [35], rocking-chair training [48], Nintendo Wii exercise [38], or a combination of different exercise forms [10, 32–34, 36, 39, 49, 52]. The remaining seven studies included different components, e.g. exercise and multidisciplinary check-ups [51], exercise and comprehensive information on, e.g. medication, nutrition, removing home hazards [17, 40, 41], exercise and a social activity programme [45], exercise and nutrition [46], and exercise and vitamin D [55]. The eight remaining studies did not include any practical exercise component. These studies included, senior meetings or discussion groups and home visits [37, 43, 44], a discussion group, activity groups and an individual intervention [8], case-management [18], anonymous self-care telephone calls [16], physical activity counselling [50], or an education programme for home-visitors [53].

Regarding modes of delivery, six studies were individually based [16, 18, 34, 40, 48, 49], seven were group-based [10, 32, 33, 35, 38, 47, 53], and 14 studies included group and individual interventions [8, 17, 36, 37, 39, 41, 43–46, 50–52, 55]. Studies including only individually based interventions were provided at home and were either self-managed [48], supervised [18, 34, 40], telephone-based [16] or digital [49]. Studies including only group-based interventions were delivered in the format of exercise groups [10, 32, 33, 35, 38, 56] or an educational group [53]. Studies including both group formats and individual interventions included group formats and home visits [8, 17, 37, 41, 43, 44], group formats and home training [17, 36, 39, 41, 52] group formats and individual counselling on health [8, 45, 46, 50, 51].

The number of sessions included in the interventions varied, as did the duration. For individually-based interventions, the number and duration of sessions ranged from one single home visit [43, 44] or one personal counselling session on nutrition [46] to daily independently performed exercise sessions (5–15 repetitions) over a period of 16 months [52]. Group-based components ranged from one single discussion group [8] to three 50 min exercise session a week for over one year [10], while the education programme for home visitors included regular education over a period of three years [53].

Studies combining group and individually-based components ranged from one single home visit and four discussion groups [43, 44] to two weekly exercise sessions over one year in combination with monthly lectures on various themes and psychosocial activities combined with a single individual geriatric assessment and counselling on fall prevention [41].

In 15 studies, the interventions were delivered by a multi-professional team [16–18, 34, 37, 40, 41, 43–46, 48, 51–53] including, e.g. physiotherapist, occupational therapists, nurses, dietitian, dentist and healthcare students. In twelve studies, the interventions were implemented by one profession, of which seven interventions were delivered by physiotherapists [32, 33, 35, 36, 38, 39, 50], one by occupational therapists [8], three by exercise instructors/leaders [10, 42, 49], and one by unspecified trained personnel [47].

### Feasibility aspects

Feasibility aspects were reported sporadically across studies. All interventions reported on methodological aspects of feasibility such as recruitment and retention/dropout numbers. With recruitment numbers, we refer to the total number of eligible participants (meeting inclusion criteria) who agreed to participate in the study. The mean recruitment rate (eligible participating population/total eligible population) in all the studies included in this review was 63%, varying from 9% [49] to 100% [33, 35, 41, 46, 52]. However, there was some inconsistency regarding how the eligible population was defined. For instance, in one study the total eligible population consisted of only those who volunteered [33], or of the population receiving an invitation [53] or the whole population in a specific community [40]. Thus, participation rates are not consistent among included interventions and this inconsistency should be taken into consideration when interpreting the mean recruitment rate. Mean retention rate in the total number of original studies included in this literature search was 85%. Retention rate varied from 37% [51] to 99% [53]. Beside the information related to recruitment and retention rates, only two feasibility/pilot studies were identified [83, 92]. Kristensson et al., investigated the feasibility of a case management intervention by specifically assessing sampling and sample characteristics as well as possible effects on perceived health [92]. Lood et al., (2016) investigated the feasibility of evaluating senior meetings in the “Elderly in the risk zone” intervention [43] among a specific group of older people (foreign-born) by specifically assessing recruitment and retention rates, questionnaire administration, and variability of data [83].

### Participants’ experiences

In relation to five of the original studies, eight related studies explored the experiences of participants [75, 76, 82, 85] or both the experiences of participants and professionals delivering the intervention [61, 62, 87, 91]. Based on qualitative methods and interviews, participants’ experiences were described related to i) a single preventive home visit (PHV) [75], ii) senior meetings [76, 82, 85, 87], iii) multidisciplinary fall prevention programmes [61, 62], and iv) case management intervention [91].

Findings from interviews on PHVs showed that home visits contributed to empowerment and increased self-esteem by making participants feel in control over their health. However, for some, it did not come at the right time, either because they felt too healthy to benefit from it or because they felt too ill to be able to participate [75]. Findings on senior meetings revealed that although independent older people may find it difficult to accept or act upon health-promoting information, the discussion groups, provided in a multi-dimensional approach, could motivate acting upon such information, and thus, senior meetings were perceived as a “key to action” [76]. These findings were in line with experiences of foreign-born older people who felt empowered by the opportunities gained, such as the possibility to meet other people, discuss experiences, as well as become acquainted with possibilities to make everyday life better and safer [82]. However, their capabilities to adhere and act upon knowledge in the long-term (six months to one year after their participation in the programme) was dependent on personal and environmental resources [85]. Furthermore, professionals delivering the interventions, revealed that for a senior meeting intervention to succeed in reaching out to the target group, it is necessary to recognise the person’s resources and empower their capabilities in maintaining health [87].

Empowerment and raised awareness were also emphasized in a group-based multidisciplinary fall prevention program delivered through a client-centered approach. The involved professionals observed that building trust and a safe atmosphere within the group increased participants’ engagement in discussions which contributed to the success of the intervention. A contributing factor for creating this sort of atmosphere was the role-shifting negotiated by the group leaders from being the expert to being a facilitator of the discussion [61, 62]. However, it was noticed that for a group format to be successful, group composition should be taken into consideration for the participants to feel fellowship [61, 82]. Furthermore, in a home-based case-management intervention, participants experienced case managers as a helping hand in navigating within the health system, and thus, contributed to feelings of control and safety [91].

Additionally, experiences of participants were explored as secondary outcomes through a survey related to a Nintendo Wii training fall prevention intervention [38], or through a single open-ended question related to a telephone-based health-promoting intervention [16]. Findings from the survey showed that training with a digital device (Wii) was experienced positively and did not lead to any adverse effect [38]. A self-care telephone intervention influenced participant’s attitudes positively, e.g. towards self-care [16].

### Effects

For several interventions, effects were evaluated in relation to a wide range of outcomes, and all, besides one intervention on nutritional counselling [46], reported a positive effect on at least one health outcome evaluated in comparison to a control group. However, the magnitude of effects and follow-ups at which interventions were evaluated, varied substantially and therefore, should be taken into consideration when evaluating effects. To summarise intervention effects, we classified health outcomes in broader categories (Table 3). For example, Balance confidence, Balance performance, Dynamic balance, Impaired balance, Postural balance, Postural sway, Velocity moment in standing balance, are categorised under “Balance”. Details on effects are found in Table 2.

**Table 3:** Overview of evaluated health outcomes of included studies in the field of health-promoting and preventive interventions for community dwelling older people in the Nordic countries from inception to 2019

Categories of health outcomes	Health outcomes evaluated	Nr. of studies evaluating the specific health outcome ^**a**^	Interventions reporting significant effects
ADL/IADL/occupational engagement	ADL, Autonomy and participation, Functional ability, Functional tasks, IADL, Leisure engagement, Leisure activities in general/physical leisure activities, Self-perceived function/Basic function/Overall function, Sense of Coherence	20	[7, 8, 34, 47, 57, 59, 66, 81, 86, 89]
Balance	Balance confidence, Balance performance, Dynamic balance, Impaired balance, Postural balance, Postural sway, Velocity moment in standing balance	12	[10, 32, 34, 39, 48, 51, 65, 101]
Bone density	BMC total body, BMD Arm/Femoral neck/Lumbar spine/Trochanter	2	[33]
Falls related parameters	Fall rates, Fall-induced injuries, Falls incidence, Falls risk, Fear of falling, Fractures rates, Incidence of falls requiring medical treatment, Lower extremity fractures, Number of fractures, Rate of injured fallers/injurious falls	18	[17, 34, 38, 40, 55, 60, 64, 69, 71, 73, 77]
Frailty and frailty indicators/morbidity	Bodily pain, Disability, Frailty e.g., tiredness in daily activities, endurance, functional balance, walking speed), Functional decline, Health transition over time, Morbidity, Mortality, Progressive decline	7	[34, 57, 77, 78, 103]
Healthcare utilisation	Healthcare consumption, Admission into long-term institutional care, Emergency department visits not leading to hospitalization, Nursing home admission.	4	[93, 103]
Health-related quality of life	HRQoL, satisfaction with physical health/psychological health, Self-rated health	10	[7, 39, 49, 59, 66, 78]
Mental wellbeing	Depressive symptoms, Discomfort/symptoms, Distress, Guidance/attachment, Life satisfaction, Likelihood of depression, Loneliness, Melancholy, Mental health, Motivation/Intrinsic motivation, Social integration, Social network, Social participation, Volition/action and coping planning	10	[16, 35, 45, 47, 66, 79, 88]
Mobility	Advanced mobility, Basic mobility, Climbing stairs, Gait/fast speed in velocity/cadence, Habitual walking speed, Lower extremity function, Maximal walking speed, Mobility performance, Outdoor walks, Reaction time of step execution, Mobility restriction, Upper extremity function, Walking duration	22	[32, 33, 35, 38, 50, 51, 57, 59, 66, 94, 97, 100, 101]
Muscle strength	Biceps strength, Carrying heavy loads, Centre of pressure velocity, Grip strength, Isokin. Knee Ext. 60 °/180 °/s, Isokin. Knee Flex. 60 °/180 °/s, Isom. Trunk Ext./Flex., Leg extensor force, Leg press, Leg strength, Maximal knee extension strength, Maximal voluntary contraction strength, RFD	13	[32, 33, 38, 48, 66, 68, 74, 95, 99]
Physical performance	Physical performance, Aerobic capacity, Physical activity level, RMR, Self-perceived physical condition	5	[94, 95]

Notes: ^a^Sum of original (first published RCT) and related studies

*Abbreviations*: *ADL* Activities of daily living, *IADL* Instrumental activities of daily living, *BMC* Body mineral content, *BMD* Bone mineral density, *HRQoL* Health related quality of life, *RFD* Rate of force development, *RMR* Resting metabolic rate

### Cost-effectiveness

Four studies presented a health-economic evaluation. Three studies adopted a cost-effectiveness analysis method [13, 72, 102] and one a cost-utility analysis method [90]. Two studies provided an economic evaluation of single interventions; a case-management intervention [90] and an education programme for home visitors [102]. The other two studies compared different interventions focused on health promotion [13], and falls prevention [72]. In these four studies, a societal perspective was chosen including cost from different sectors e.g., health care and social care. The time horizon used varied from three months [13], one year [13, 90], two years [72] and up to three years [102]. All studies based their estimates of costs on intervention costs, healthcare costs and municipality costs. In addition, the value of informal care was included in one study [90]. Cost-effectiveness was evaluated in relation to active life-years gained [102], quality-adjusted life-years (QALYs) [13, 90] and number of injurious falls prevented [72].

Findings from the economic analysis showed that two interventions were considered cost effective [13, 72] whilst two were not [90, 102]. A one-session discussion group was found to be more cost-effective when compared to an individual intervention or an activity group in an intervention comparing three different occupation-focused health-promoting interventions to a control group [13]. The discussion group showed significant effects on QALYs gained at 3 and 12 month follow up’s and lower total costs [13]. Furthermore, an exercise intervention showed high probability to be cost-effective in preventing falls in relation to a threshold of 3000 euro per injurious fall prevented when compared to three other fall preventive interventions focusing on exercise and vitamin D supplements [72]. In contrast, no significant difference was observed in total costs or QALYs gained when comparing a case management intervention to no intervention in a cost-utility analysis. Nevertheless, the case management intervention led to lower levels of informal care and need for help with instrumental ADLs [90]. Neither did a training programme for home visitors result in significant differences in total cost or active life-years gained in comparison with usual practice of performing preventive home visits [102].

## Discussion

This scoping review provides a comprehensive overview of health-promoting and preventive interventions for community-dwelling older people in the Nordic countries that to some extent, can guide decision-making in a Swedish municipality context. However, while all included studies report some positive effects, not all potentially effective interventions can be implemented since resources are limited. Thus, the evidence on effects needs to be critically reflected upon, but several other factors need to be considered as well. Our study exposes gaps in knowledge regarding cost-effectiveness, experiences of participants and feasibility of the interventions, knowledge that could broaden the understanding of which interventions seem most promising and feasible to implement from a decision-makers´ perspective.

While the scope of this review includes interventions with different foci, the summary of findings on the seven evaluated factors, show that some interventions such as senior meetings, preventive home visits (PHV) and exercise interventions alone or combined with other components, seem to be strong candidates for implementation, e.g. [10, 43, 50]. In all, the total evidence for these interventions included positive effects on a range of outcomes, in some cases confirmed by evaluations at different follow-ups, with established cost-effectiveness, and supported by qualitative findings based on the experiences of participants.

In the section below we provide a deeper discussion about the previously mentioned intervention examples and argument how the findings from this review could guide decision making and how additional knowledge, generally missing across the different interventions, is needed to better guide decisions on which interventions to implement.

Senior meetings, one type of intervention investigated in four different studies, seems potentially effective in promoting general health and wellbeing among community-dwelling older people [8, 37, 43, 44]. The study which provides the broadest evidence base is the “Elderly Persons in the risk zone”-study conducted in Gothenburg [43], which evaluated a four-sessions senior meeting intervention combined with a home visit. Several related studies support the implementation of senior meetings given the positive results on a range of health outcomes, e.g., physical function [77] and ADLs [81], outcomes for which effects were established at different follow-ups (3 months to 2-year follow-ups). Qualitative findings on the experiences of participants also provide an understanding of why the intervention was effective by concluding that senior meetings were experienced as a “key to action” in empowering participants to engage in preventive approaches to improve health [76]. The benefits of senior meetings, albeit with other content, were also verified in the studies by Zingmark et al., [8] and Johansson et al., [37]. In the study by Zingmark et al., [8] two group-based formats of interventions (a discussion group and an activity group) were implemented by occupational therapists which both resulted in positive effects. In our results, evidence on cost-effectiveness regarding senior meetings was limited to the study by Zingmark et al., who found a one-session discussion group to be the most cost-effective intervention format [13]. Recently, however, a publication based on data from the “Elderly Persons in the risk zone” supports the cost-effectiveness of senior meetings as well, even in the long term (over four years) [109]. Thus, senior meetings seem to be a strong candidate for implementation in a Swedish municipality context. Yet, the exact format can be further discussed given the variation in the number of sessions and the specific content, e.g. one session discussion [8], four sessions combined with a home visit [43], twelve sessions combined with two home visits [37]. In addition, feasibility aspects related to recruitment during implementation in a municipality context seem to be a critical feature to improve reach in the intended population, thus requiring specific contextual knowledge [110].

Our results show that PHVs have the potential to improve general health by preventing deterioration in health in community dwelling older people. However, PHVs have varied regarding the specific format e.g. from one visit [43] to twelve visits [18] and have shown positive effects on several outcomes e.g. limiting progression in morbidity [78], reducing the number of emergency department visits [18], maintaining ADL ability [8] reducing lower extremity fractures [40]. Positive effects were also reported for an education programme for the home visitors conducting the PHVs, in terms of lower admission rates to nursing homes for those receiving two home visits per year [103]. The most promising results on PHVs were established in the “Elderly Persons in the risk zone” study where a single home visit was evaluated and showed positive effects ADLs [81], frailty and fear of falling [77], life satisfaction and morbidity [78]. This study was the only one, among PHV interventions, to conduct a 2-year follow up at which some effects persisted and thus validates post-intervention effects [81]. The positive effects of PHVs in the “Elderly Persons in the risk zone” study are partly explained by the experiences of participants, who felt empowered and in control as a result of the information given and having the opportunity to discuss health-related matters with a qualified professional [75]. However, these findings on long-term effects are in contrast to a previous PHV trial that indicated that intervention effects remained only for as long as the home visits were ongoing [111], and thus, highlights the importance of long term follow-ups over. Conflicting results regarding specific effects of PVHs and their health-economic effects have been reported also in a recent report from SBU Enquiry Service (Swedish Agency for Health Technology Assessment and Assessment of Social Services) about preventive home visits, also referred to from the Swedish National Board on Health and Welfare [112]. In some studies, though, PHVs have shown to be cost-effective while annual follow-up visits can be potentially even more cost-effective. Such findings have been established when conducting health economic analysis based on data from the Elderly Persons in the risk zone [109] as well as in a previous Swedish study including twice-annual home visits over a period for two years [111]. Despite the conflicting results on some outcome effects of PHVs [113], they still can be considered a good alternative to group-based interventions, e.g. senior meetings, since not all potential participants can or like to engage in a group format.

Interventions including exercise or combining exercise with other components (e.g. medication review, guidance on nutrition, cessation of alcohol and smoking, home hazard assessment and modifications) showed to be promising for preventing falls. Findings on these interventions showed improvements in different factors related to falls risk and physical functioning, e.g. muscle strength, mobility, balance or self-rated health [34, 35, 38, 59] which could indirectly lead to fall reduction [114]. Positive effects were observed for both home-based [34] and group-based interventions [39], regardless of whether they were shorter (3 months) [35] or longer (1 year) in duration [33]. Furthermore, interventions including more frequent group sessions reported additional effects, such as improvement in motivation to continue with physical activity [36, 47], and perhaps consequently a reduction in injurious falls and fractures, as reported in two fall prevention interventions [10, 17]. Both interventions included balance exercise in combination with resistance/strength exercise provided over one year or longer, but varied in terms of content, number of sessions, and delivery approaches used e.g. multifactorial [115] and multiple components [17]. In line with evidence from a recent systematic review and meta-analysis, exercise-based interventions, aiming to improve balance and strength, are one of the most feasible and cost-effective approaches to prevent falls among older people living in the community [114]. This approach has also been integrated into some current Swedish guidance, on physical training, balance and more, issued from the National Board on Health and Welfare in the form of training for professionals working with older people and fall prevention [116]. However, effectiveness of exercise-based interventions is dependent on the uptake and long-term adherence [117]. Groups sessions led by professionals over a longer period (1 year or more) seems to affect this aspect positively but can be costly, foremost in terms of human resources needed if provided to a large population of older people. Since group training might not be the solution for all, other effective alternatives such as multifactorial interventions could work in these cases. Also, multifactorial interventions have shown positive effects on preventing falls [118] and could be considered an alternative to exercise-based interventions. Nonetheless, no health-economic evaluation was identified for these interventions, and thus, still makes them less robust in terms of cost-effectiveness.

While our results, indicate that there are several health-promoting and preventive interventions that could improve health and well-being among community-dwelling older people, implementation needs to be considered, not only in relation to effects but also concerning the resources available, i.e. how limited resources can be used in a way that yields the largest health benefits [20, 21] and other feasibility aspects such as reach in the population; a key factor for successful implemtation of research in practice.

Health economic evaluations, including evaluation of both costs and effects, can provide such important information. However, in this scoping review, only four health economic evaluations were identified, indicating a general lack of information to guide decision making. However, information regarding intervention content, e.g. duration and intensity of interventions, can at least provide some information about the resources required. Regarding individual interventions, the study by Dahlin-Ivanoff et al. included one single preventive home visit requiring one and a half to two hours of a professional’s time [43] in contrast to the study by Möller et al. in which a case management intervention, required at least one hour per month during a 12-month intervention of professional’s time [18]**.** Similarly, for group-based interventions, the span for the time required was two hours for a one session discussion group [13], to two and a half hours per week over the course of one year [10]. While these examples all include interventions with some positive effect, the time for which staff need to be allocated differs substantially. Even though these examples lack information on other types of costs that can be affected by interventions (e.g. social care consumption), they provide some guidance on which resources are needed and the magnitude of staffing which is a central cost of a health-promoting or preventive intervention [13].

Despite a growing literature of health-promoting and preventive interventions that have shown positive effects in well-controlled trials, the translation of such trials to practice has proven to be challenging [20]. Evidence has shown that feasibility or pilot studies are important to ensure effective practical implementation and to decrease threats to validity of health outcomes [119]. However, in our literature search, there was a lack of piloting and feasibility studies. In the absence of feasibility or pilot studies, other reported aspects such as information on study participation rates and adherence could indicate the degree to which an intervention reaches out to the target population, and thus, increase chances of a successful translation of research evidence into clinical practice [119]. Reaching older people with health promotion is crucial for achieving a health impact for the whole population, but has also been shown to be challenging [110, 120]. Findings from all 27 original studies, in this review, showed that approximately a third of the persons eligible declined to participate due to different reasons, i.e. being too sick or too healthy [75]. Qualitative data on experiences of participants could to some extent reveal why an intervention is or is not appealing to larger groups of older people, however, only a few studies on experiences of participants were identified in this review.

While this review provides some guidance on which interventions have shown positive health effects in a Nordic context, future research is needed on how to translate evidence into practice, e.g. through exploring alternative ways of reaching out to a larger population and incorporating support for behaviour change and adherence in the long-term. Some examples of new promising approaches explored in this review were Wii training [38] and physical activity counselling [50]. The digital approaches used through video training or self-care telephone calls are potentially feasible to be implemented considering the more limited resources required to implement them, e.g. the smaller number of direct personal contacts needed with providers of health care for older people while still resulting in positive effects. In light of the ongoing coronavirus pandemic and related measures of social distancing, the importance of addressing loneliness and isolation among older people is accentuated. Digital approaches to delivering effective interventions could complement the challenge of isolation and the need to reach out to a higher number of older people. For example, using smartphones and tablets may be a potentially cost-effective way to increase reach in the population. At present, there is a big supply of smartphone applications for exercise, however, most lack evidence regarding their scientific and implementation validity in the older population. Research in the area is, however, developing and one example is an ongoing large clinical trial on digital fall prevention in Sweden [121]**.**

Finally, in discussing the results of this study, it is notable that some important aspects of healthy ageing, were less frequently evaluated. Only two studies focussed on mental wellbeing and social participation, one showed some effects in reducing loneliness [45] and the other in improving general mental health [16]. This gap in research has also been supported in other reviews, where promoting wellbeing and mental health have shown to be both effective and potentially cost-effective [122, 123], and should, therefore, be further researched.

### Strength and limitations

The scope of this review was broad. It included information on several factors extracted from all identified original and their related studies, and therefore provides an overview of the knowledge base in the field of health-promoting and preventive interventions in the Nordic countries. Given the broad scope of this review, we choose to not include some information, e.g. data concerning when studies were performed or adverse events, which could be seen as a limitation of the study.

Data concerning when studies were performed would enrich information on the context and content of the interventions. However, the description of the study period, e.g. the period for the recruitment of participants, have not been reported consistently among all studies, therefore might not have produced many data. Although a wide range of outcome effects was extracted, important information on adverse events was not extracted and beyond the scope of this study, guided primarily by the MRC guidelines. Additionally, recent systematic reviews show that adverse events, for example, concerning fall prevention programmes seem to be rather poorly reported hence, would probably not make a significant difference in our conclusions, if included in the analysis [12, 118]. Another important factor to consider, which may lead to better developed and evaluated interventions, is if the studies have a theoretical foundation that may explain the causal link between intervention and outcomes [28]. However, considering the already broad focus of this review, we choose to limit the presentation of results and not include data on the theoretical foundations for each intervention. Furthermore, the quality of the included studies has not been evaluated the same way it would be assessed in a systematic review, meaning that the quality can differ between the studies. It is, however, in line with PRISMA guidelines on scoping reviews considering this step optional [27]. Yet a quality assessment of the included studies or grading of evidence might have led to stronger conclusions as a result of a reduction in uncertainty related to outcome effects.

Finally, this review did not include studies from the rest of the world, albeit such studies could have provided relevant information. The choice to do so was due to the importance of contextual factors concerning complex interventions [124]. Limiting the inclusion of interventions deriving from countries with similar welfare models and cultural context might increase chances of effective implementations of promising interventions. Furthermore, research shows that there is is often a lack of information regarding the influence of the context when conducting and evaluating complex interventions [124]. Thus, more research on the influence of contextual factors in the effectiveness of certain interventions would add to the knowledgebase important for decision-makers.

## Conclusions

This scoping review, following the MRC guidelines, provides an overview of the evidence and evidence gaps of health-promoting and preventive intervention studies for community-dwelling older people in Nordic countries hence, of importance for decision-makers, research councils and researchers.

All interventions, besides one, showed positive effects on at least one health outcome, although the magnitude of effects and number of follow-ups differed substantially. Given that evidence on effects alone are not enough information for decision-makers, information on other factors is needed. Overall, there was a general lack of studies related to cost-effectiveness, experiences of participants and feasibility. Therefore, such studies are strongly warranted. In all, based on the evidence presented, senior meetings, preventive home visits and exercise interventions alone or combined with other components seem to be strong candidates for implementation in a Swedish municipality context.

## Supplementary information

**Additional file 1.** Search strategies and numbers of records identified in each database.

**Additional file 2.** Preferred Reporting Items for Systematic reviews and Meta-Analyses extension for Scoping Reviews (PRISMA-ScR) Checklist.

## Data Availability

All data analysed during this study are included in this published article and its additional files. The search strategy is available in Additional file 1. PRISMA extensions for scoping reviews-checklist is included in Additional file 2.

## Abbreviations

WHO: World Health Organisation
PRISMA-ScR checklist: Preferred Reporting Items for Systematic reviews and Meta-Analysis extension for Scoping Reviews
MRC guidelines: Medical Research Council guidelines
PICO: Population, Intervention, Comparison, Outcome
HP: Health promotion
PP: Primary prevention
RCT: Randomised controlled trial
CONSORT: Consolidated Standards of Reporting Trials
PHV: Preventive home visits
QALYs: Quality adjusted life years
ADL: Activities of daily living
