# Supplementary material for: Health-promoting and preventive interventions for community-dwelling older people published from inception to 2019: a scoping review to guide decision making in a Swedish municipality context
Source: Arch Public Health. 2020 Oct 14;78:97. doi: 10.1186/s13690-020-00480-5 (PMC7556574; doi:10.1186/s13690-020-00480-5)
Supplement: Supplementary file 1 — Additional file 1. Search strategies and numbers of records identified in each database. [file 13690_2020_480_MOESM1_ESM.docx]

**Search strategies and numbers of records identified in each database**Date of searches for PubMed and SCOPUS: 2^nd^ January 2019 9^th^ of January 2019

**PubMed**

1. MESH Terms Health promotion (n=70552)
2. [Tittle/Abstract] prevention OR intervention OR “health programme” OR “health program” OR “health education” (n=1030206)
3. 1 OR 2 (1068830)
4. MESH Aged (n=2889493)
5. [Tittle/Abstract] (“old people” OR aged OR “old population” OR senior OR elderly OR “older adults” OR “well older people” OR “senior citizen” OR “old age” OR “advanced age” OR geriatric OR ageing OR aging) (n=941406)
6. 4 OR 5 (n=238634)
7. [Tittle/Abstract] (“independent living” OR “community dwelling” OR “home dwelling” OR “community living” OR “living alone” OR “ageing in place”) (n=27785)
8. 3 AND 6 AND 7 (n=4482)
9. “nordic countries” OR sweden OR norway OR finland OR iceland OR denmark OR “faroe islands”
10. 8 AND 9 (n=351)
11. “randomized controlled trial” OR “random allocation” (n=692189)
12. 10 AND 11 (n=167)

**Scopus**

**When adding health promotion**

health promotion OR TITLE-ABS-KEY (health promotion OR prevention OR intervention OR "health programme" OR "health program" OR "health education" ) AND TITLE-ABS-KEY("old people" OR aged OR "old population" OR senior OR elderly OR "older adults" OR "well older people" OR "senior citizen" OR "old age" OR "advanced age" OR geriatric OR ageing OR ageing) AND TITLE-ABS-KEY("independent living" OR "community dwelling" OR "home dwelling" OR "community living" OR "living alone" OR "ageing in place") AND ("nordic countries" OR sweden OR norway OR finland OR iceland OR denmark OR "faroe islands") AND ("randomized controlled trial" OR "random allocation")

(n=446)

EXCLUDE ( SUBJAREA,"BIOC" ) ) AND ( EXCLUDE ( DOCTYPE,"re" ) OR EXCLUDE ( DOCTYPE,"ch" ) ) AND ( EXCLUDE ( EXACTKEYWORD,"Middle Aged" ) OR EXCLUDE ( EXACTKEYWORD,"Dementia" ) OR EXCLUDE ( EXACTKEYWORD,"Alzheimer Disease" ) ) AND ( LIMIT-TO ( AFFILCOUNTRY,"Sweden" ) OR LIMIT-TO ( AFFILCOUNTRY,"Finland" ) OR LIMIT-TO ( AFFILCOUNTRY,"Norway" ) OR LIMIT-TO ( AFFILCOUNTRY,"Denmark" ) OR LIMIT-TO ( AFFILCOUNTRY,"Iceland" )

(n=162)

Date of searches for EBSCO (CINAHL, Academic search elite, PsycINFO, SocINDEX, SPORTDiscus: 9^th^ of January 2019

**EBSCO (CINAHL, Academic search elite, PsycINFO, SocINDEX, SPORTDiscus)**

Limiters: English Language; Human; Randomized Controlled Trials; Age Groups: Aged: 65+ years; Publication Type: Peer Reviewed Journal; English; Expanders: Apply related words; Search modes: Find all my search terms

1. “Health promotion” (n=83,175)
2. TI (prevention OR intervention OR “health programme” OR “health program” OR “health education”) (n=170,199)
3. AB (prevention OR intervention OR “health programme” OR “health program” OR “health education”) (n=814,880)
4. TI (“old people” OR aged OR “old population” OR senior OR elderly OR “older adults” OR “well older people” OR “senior citizen” OR “old age” OR “advanced age” OR geriatric OR ageing OR aging) (n=209,226)
5. AB (“old people” OR aged OR “old population” OR senior OR elderly OR “older adults” OR “well older people” OR “senior citizen” OR “old age” OR “advanced age” OR geriatric OR ageing OR aging) (n=674,511)
6. TI (“independent living” OR “community dwelling” OR “home dwelling” OR “community living” OR “living alone” OR “ageing in place”) (n=8,563)
7. AB (“independent living” OR “community dwelling” OR “home dwelling” OR “community living” OR “living alone” OR “ageing in place”) (n=29,056)
8. “nordic countries” OR sweden OR norway OR finland OR iceland OR denmark OR “faroe islands” (n=704,209)
9. 1 OR 2 OR 3 (n=911,466)
10. 4 OR 5 (n=707,382)
11. 6 OR 7 (n=31,564)
12. 8 AND 9 AND 10 AND 11 (n=361) (Academic search elite=197; CINAHL=26; PsycINFO=88; SocINDEX=22; SPORTDiscus=28;)
